# Supplementary material for: Factors associated with the presence and intensity of ongoing symptoms in Long COVID
Source: PLoS One. 2025 Apr 23;20(4):e0319874. doi: 10.1371/journal.pone.0319874 (PMC12017833; doi:10.1371/journal.pone.0319874)
Supplement: S1 File — The 46 included symptoms that were derived from a comprehensive review of systems modeled on the WHO’s Global COVID-19 Clinical Platform Case Report Form for Post COVID Condition. (DOCX) [file pone.0319874.s001.docx]

**S1 File**

The following symptoms were assessed: 1) balance or walking problems, 2) not being able to move and/or feel one side of body or face, 3) chest pain, 4) constipation, 5) Diarrhea, 6) dizziness or light-headedness, 7) fainting of blackouts, 8) fatigue, 9) fever, 10) hallucinations, 11) headaches, 12) jerking of limbs, 13) joint pain or swelling, 14) loss of appetite, 15) nausea or vomiting, 16) numbness or tingling, 17) pain on breathing, 18) palpitations or heart racing, 19) persistent cough, 20) problems hearing, 21) persistent muscle pain, 22) pain or fatigue after exercise, 23) problems passing urine, 24) problems seeing, 25) problems swallowing, 26) ringing in ears, 27) shortness of breath, 28) skin rash or changes in color, 29) slowness of movement, 30) sleeping less, or difficulty falling asleep, 31) stiffness of muscles, 32) tremors, 33) trouble concentrating, and 34) weakness in arms or legs, 35) unusual behavior or change in personality, 36) painful menstrual periods, 37) erectile dysfunction, 38) falling, 39) forgetfulness or “brain fog”, 40) loss of control of bladder, 41) reduced smell, 42) reduced taste, 43) seizures, and 44) sleeping more.
