# Supplementary material for: Factors associated with the presence and intensity of ongoing symptoms in Long COVID
Source: PLoS One. 2025 Apr 23;20(4):e0319874. doi: 10.1371/journal.pone.0319874 (PMC12017833; doi:10.1371/journal.pone.0319874)
Supplement: S2 File — A more elaborate explanation of the statistical analyses used. (DOCX) [file pone.0319874.s002.docx]

**Appendix B**

*Factor analysis to establish symptom intensity for PASC symptoms*

To identity groupings of symptoms that address PASC in the World Health Organizations Global COVID-19 Clinical Platform Case Report Form for Post COVID Condition, we performed a factor analysis and 2-parameter logistic model based on item response theory to develop a measurement for symptom presence and intensity (Table 2). We performed an exploratory factor analysis (EFA) with oblimin rotation to identify groups of symptoms that seem to be measuring the same underlying construct and measure the loading of items onto each factor to identify redundant items. All items with a factor loading below 0.3 in EFA were excluded using a stepwise approach. We found that most symptoms grouped together into a single factor. Reduced smell and reduced taste grouped together into a separate factor. We omitted unusual behavior or change in personality and erectile dysfunction because they did not contribute to either symptom grouping (factor loading <0.3). The remaining items were moved to confirmatory factor analysis (CFA) to verify the factor structure, and we removed items with a factor loading below 0.3 in CFA and high modification indices. We omitted sleeping more due to its low contribution to the construct of persistent symptoms (factor loading <0.3), and we omitted forgetfulness (or “brain fog”) based on the high modification index (>80) with trouble concentrating (also deemed part of “brain fog”) and it having a lower factor loading. The remaining items were moved to an Item Response Theory (IRT) based 2-parameter logistic (2-PL) model to determine how well a symptom can identify patients with different symptom intensities and to calculate a population-based t-score with a gaussian distribution. The IRT based 2-PL model confirmed adequate symptom selection. We then performed a differential item functioning (DIF) analysis to assess whether certain demographics (e.g., age, gender, level of education, race) influence the probability of having a certain symptom *while the true underlying symptom intensity would otherwise be the same*, and therefore introduce bias in the symptom severity score. For example, men will answer questions about erectile dysfunction differently than women, and thus such an item should be removed. We performed DIF analysis using the GPCMLasso and lordif packages in RStudio (version 2022.12.0 Build 353, R version 4.2.2), and items flagged for DIF were excluded. We omitted painful menstrual periods (only relevant for women), falls (older patients fell more), loss of control of bladder (more common for women), and seizures (older patients had more seizures). We then repeated CFA and IRT and found good fit statistics. The selected symptoms that measure the same construct were converted into a t-score resembling PASC symptom presence and intensity, which was used as our primary outcome. The second factor, constituting reduced taste and reduced smell, was used as secondary outcome for an unplanned secondary analysis.

*Statistical analysis*

Descriptive statistics were performed for all patient characteristics. We used parametric and non-parametric bivariate analyses depending on data distribution to seek factors associated with the long COVID symptom presence and intensity scale, loss of taste, and loss of smell. All variables with a *P*-value of below 0.10 in bivariate analysis were moved to multiple linear and logistic regression to assess independent associations. We addressed multicollinearity of the mental health measures (PHQ-9, GAD-7, and PC-PTSD-5)^25^ by running separate models with one mental health measure at the time, selecting the model with the lowest Akaike Information Criterion (AIC) and highest R^2^ (model with GAD-7 alone) and omitted other mental health measures from the analysis (PHQ-9 and PC-PTSD-5). All *P*-values below 0.05 were considered statistically significant. Data were analyzed using RStudio (version 2022.12.0 Build 353, R version 4.2.2) and Stata 13.0.
 A priori power analysis indicated that 124 participants would provide 0.80 statistical power to detect a correlation of 0.25, with alpha set at 0.05. This means that a mental health measure would account for 6% of the variation in the long COVID symptom intensity scale. In addition, previous simulation study indicates that our study should obtain excellent reliability for exploratory factor analysis (assuming a reliability criterion 0.98, a variable to factor ratio of 5:1 and a 3-factor solution) with 200 participants assuming wide communality (factor loading between 0.2 and 0.8)^26^.
