# Supplementary material for: Factors associated with the presence and intensity of ongoing symptoms in Long COVID
Source: PLoS One. 2025 Apr 23;20(4):e0319874. doi: 10.1371/journal.pone.0319874 (PMC12017833; doi:10.1371/journal.pone.0319874)
Supplement: S3 File — The formula that can be used to generate the PASC symptom presence and intensity scores. (DOCX) [file pone.0319874.s003.docx]

**S3 File.** Conversion table for the PASC symptom presence and intensity score.

| **Summed score** | | **F1 score** |
| --- | --- | --- |
| 0 | -2.263597392 | |
| 1 | -1.906762913 | |
| 2 | -1.622938280 | |
| 3 | -1.388506372 | |
| 4 | -1.188212550 | |
| 5 | -1.012490775 | |
| 6 | -0.855028745 | |
| 7 | -0.711454680 | |
| 8 | -0.578619076 | |
| 9 | -0.454175897 | |
| 10 | -0.336325220 | |
| 11 | -0.223647995 | |
| 12 | -0.114995970 | |
| 13 | -0.009416046 | |
| 14 | 0.093903165 | |
| 15 | 0.195670208 | |
| 16 | 0.296520044 | |
| 17 | 0.397037043 | |
| 18 | 0.497774163 | |
| 19 | 0.599270203 | |
| 20 | 0.702066896 | |
| 21 | 0.806727610 | |
| 22 | 0.913859561 | |
| 23 | 1.024141575 | |
| 24 | 1.138359444 | |
| 25 | 1.257450885 | |
| 26 | 1.382562572 | |
| 27 | 1.515123635 | |
| 28 | 1.656945582 | |
| 29 | 1.810369675 | |
| 30 | 1.978500086 | |
| 31 | 2.165584309 | |
| 32 | 2.377632363 | |
| 33 | 2.623343383 | |
| 34 | 2.915098099 | |

**Formula**: t-score = (F1 * 10) + 50
