# Supplementary material for: Factors associated with the presence and intensity of ongoing symptoms in Long COVID
Source: PLoS One. 2025 Apr 23;20(4):e0319874. doi: 10.1371/journal.pone.0319874 (PMC12017833; doi:10.1371/journal.pone.0319874)
Supplement: Attachment — Submitted filename: Response to reviewer comments - v2.docx [file pone.0319874.s006.docx]

**Response to reviewer comments:**

| *Reviewer comment* | *Author response* |
| --- | --- |
| The article addresses a relevant and globally significant public health issue. However, I believe some parts could be adjusted. | Please see edits throughout. |
| The title and the short title seem to belong to different articles, as they are not consistent with each other. For this reason, it is unclear what the focus of the article will be. | Curious that you find them unrelated? We would appreciate some elaboration if possible.  The title and subtitle should reflect the applicable knowledge from the experiment, and—in our view—they do. The subtitle refers to the most important findings related to the factors associated with symptom presence and intensity ascribed to PASC (symptoms of anxiety was the strongest correlate). |
| Regarding the objective, it could be written in a more specific way that clearly reflects the purpose of the study, as it is currently confusing what the study aims to achieve. This is compounded by the fact that the titles differ. | We’ve added a sentence elaborating on the study’s purpose (line 129-131):  “The purpose of this study was to identify which factors, including psychosocial aspects of health, are associated with symptom presence and intensity ascribed to PASC.” |
| I think it is important that the introduction clearly states the purpose of the study, as it mentions the identification of factors associated with the presence and intensity of ongoing symptoms in Long COVID, but then it discusses anxiety and depression (are these the identified factors?). This is somewhat confusing. Similarly, in the results section, anxiety factors and symptoms are mentioned first. | Please see edits in the manuscript to further elaborate on the study purpose and the emphasis on mental health.  In the results, symptoms of anxiety are mentioned first as they turned out to be the strongest correlate of symptom presence and intensity ascribed to PASC. We chose to describe the most important / strongest correlates first as the order would otherwise be random. The order of mentioning the correlates does otherwise not matter.  We’ve also added the following references in the edits:  Castanares-Zapatero D, Chalon P, Kohn L, Dauvrin M, Detollenaere J, Maertens de Noordhout C, et al. Pathophysiology and mechanism of long COVID: a comprehensive review. Ann Med 2022;54:1473. https://doi.org/10.1080/07853890.2022.2076901. |
| Although the discussion mentions and clarifies associated factors, the relationship with anxiety remains unclear. | Acknowledged. Please see edits in the discussion. |
| Finally, in the conclusion, the study does not seem to answer the stated objective (to identify associated factors), as it only discusses anxiety factors and symptoms as the sole associated factors. | Acknowledged. Please see edits in the conclusion. |
